# Are Chinese Entrepreneurs Happier than Employees? Evidence Based on a National Workforce Survey in China

**DOI:** 10.3390/ijerph18010179

**Published:** 2020-12-29

**Authors:** Chang-Lan Xia, Tung-Ju Wu, An-Pin Wei, Pei-Guan Wu

**Affiliations:** 1 International School of Business and Finance, Sun Yat-sen University, Zhuhai 519082, China; xiachlan@mail.sysu.edu.cn (C.-L.X.); weianp@mail.sysu.edu.cn (A.-P.W.); 2 School of Management, Harbin Institute of Technology, Harbin 150001, China; tjwu@hit.edu.cn

**Keywords:** job-related wellbeing, employee, entrepreneur, subsistence entrepreneurship

## Abstract

Most studies consider entrepreneurship in Chinese a happier career choice, while the adverse effects of entrepreneurship on wellbeing have been overlooked. In this research, the effect of career choice on job-related wellbeing is explored using multiple indicators. Differences in the career choices of employees and entrepreneurs are examined in the first section of the study, and the motives for entrepreneurship are studied in the second section. Job-related wellbeing is regarded as consisting of job satisfaction, subjective wellbeing, and physical wellbeing. The data were obtained using the Chinese Labor-Force Dynamic Survey, and the sample consisted of 6108 employees and 2075 entrepreneurs from 29 provinces and cities in China. T-test, chi square test, and ordinal logistic regression were conducted. The analysis in the first section reveals significant differences in job-related wellbeing between employees and entrepreneurs along with differences in autonomy and perceived equity. Entrepreneurs are found to be less satisfied and unhappier than employees. The heterogeneity of the motives for entrepreneurship is highlighted in the second part, and its significant role in the wellbeing of entrepreneurs is explored. Subsistence entrepreneurs have been found to constitute up to 64% of all entrepreneurs. Subsistence entrepreneurship is negatively associated with job satisfaction and subjective wellbeing.

## 1. Introduction

Chinese IT employees report that “rule 996” is currently widespread in the industry and has become a factor in career development decisions, i.e., choosing between searching for a job or becoming an entrepreneur [1]. Rule 996 states that an employee should work from 9 am to 9 pm 6 days per week. This has led to heated debate, as it is not only widespread in the IT industry, but also in many companies in other sectors. The attainment of job-related wellbeing or happiness is severely impeded by the application of rule 996 in the working environment, and has thus led Chinese youths to believe that it is more satisfying to become an entrepreneur than to work for others [1]. The Chinese government also offers support to entrepreneurs in the form of political and financial policies [2]. The supportive environment for entrepreneurs offers a more attractive career choice than the harsh working conditions experienced by the Chinese employees.

Workforce well-being and satisfaction is an important research topic when considering working conditions and public health [3,4]. The notion of job-related wellbeing, which is derived from subjective wellbeing, refers to individual wellbeing research in the work context [3,5]. Leung et al. suggest that subjective wellbeing should be separated into physical and psychological wellbeing to correspond with “body and soul” [6]. Micu and Necula also point out that all aspects of work affect individuals, including physical, intellectual, emotional, and spiritual elements, and hence job satisfaction can be viewed as a component of happiness [7]. Thus subjective wellbeing, physical wellbeing, and job satisfaction are all considered in the present study of job-related wellbeing, or happiness. Studies of the levels of satisfaction of both employees and entrepreneurs in China have been previously conducted. The Job Happiness Index Survey indicates that the average degree of happiness of Chinese employees is 2.57 out of 5, which is supported by the website of China Human Resource Development [3]. A survey of Chinese entrepreneurs tracked their degrees of happiness between 2005 and 2014, with scores between 3.46 and 3.78 out of 5 [8]. However, it is still hard to say that Chinese entrepreneurs were found to be happier than employees, because comparable evidence is lacking, as the data came from different survey systems.

The design of work can affect the mental and physical wellbeing of an individual [4,9,10]. There is evidence that entrepreneurs are much happier and satisfied than employees across Europe [11,12,13,14], America [13], and Canada [11]. Although substantial research has been devoted to proving that self-employment is a happier career path, the definition of “entrepreneur” is slightly more complex than that of “employee.” Some research didn’t agree to define people who were pushed to become self-employed without access to salaried work as “entrepreneurs” [15,16,17], while many studies had admitted them as “entrepreneurs “concerning economic effect of business [18,19]. Both pecuniary and non-pecuniary benefits can be motives for new venture creation [17]. While differences in motivations for work have a substantial effect on levels of job satisfaction, subjective wellbeing, and physical wellbeing [15,16]. Schoar suggests that there are at least two distinct types of entrepreneurs, whose economic objectives, skills, and roles in the economy vary [18]. Subsistence entrepreneurs run businesses to provide subsistence income, while transformational entrepreneurs aim to create businesses that can grow far beyond the scope of individual needs [18]; subsistence entrepreneurship is fundamentally about survival, and individuals involved in it often lack experience in deploying the cognitive skills [17]; subsistence entrepreneurs often get stuck in unproductive or small firms and achieve lower profits than transformational entrepreneurs [16].If entrepreneurs emerge not by choice but by necessity, they may not turn out to be as happy as expected [13,16,19]. Individuals who are “pushed” into self-employment due to the scarcity of jobs may experience less satisfaction or happiness [20]. Individuals who are motivated by non-pecuniary benefits will probably be less affected by unexpected financial hardship or unforeseen stress and excessively long working hours [18]. 

To explore the relationship between happiness and career choices in China, common factors influencing satisfaction levels among venture founders and employees, such as autonomy of work, are examined in this study. The motives for embarking on start-up businesses and the characteristics of ventures, which may also affect the degree of entrepreneurial happiness, are also investigated. Moreover, according to “bottom-up” perspectives related to the causal assumptions of wellbeing, a person’s wellbeing depend on external events, and broad situational and demographic factors [21]. For instance, evidence has long been accumulating concerning the association between marital status and different well-beings at all ages in the main effect model and buffering model [22]. Despite the difference of subjective stress, both models concluded that marital relationships are often consequences of different types of wellbeing, especially for older people [22]. The classic “Easterlin paradox” also shows a complex relationship between income and wellbeing and reminds us that different regions could influence the relationship aforementioned through different economic characters [22]. Work which has always been considered a pivotal aspect of life, affect wellbeing from working time and industry by the trade-off between leisure and time spent to work [23,24]. The gender difference in wellbeing results from the gender gap in life expectation, which may be influenced by education [25]. Concerning association effect, different demographic factors will be controlled in the study as well. 

The study makes several contributions to the research into Chinese happiness. First, other studies have focused on the employees rather than the entrepreneurs in the Chinese workforce. The aim of this study is to provide new evidence about the happiness of the Chinese workforce by using data from the China Labor-force Dynamic Survey. The survey covered the labor force of 29 provinces and districts, and hence provides comparable evidence about the difference between entrepreneurs and employees. Second, by establishing a theoretical and empirical basis for linking the motives of entrepreneurship to happiness and by including different types of entrepreneurship in the model, the underlying mechanism of happiness can be revealed. The study thus complements new theoretical explanations and empirical evidence concerning job-related wellbeing and the choice of career path. In the following, more details on the scientific background will be provided, include presenting hypotheses guiding our research.

### 1.1. Entrepreneurs are Expected to Be More Satisfied than Employees

The Job Demand Control Support model (JDCS) can help explain the mechanism of job satisfaction [4,21]. The model is used to assess job demand and job control dimensions and indicates a low job-related wellbeing outcome from jobs with the highest demands and lowest job control. The model suggests that job demands are determined by the job requirements and that job control involves the ability to decide to finish the job [4,26]. Molina-Sánchez et al. consider that control over activities is described as having the latitude to make decisions [4]. Higher demands are associated with lower job satisfaction and more significant anxiety, while the opposite is true for control [4,9]. The power of individual control is also related to the organization or participation in organizational decision-making [5]. Job control is closely related to authority and responsibilities [26], is also refereed as autonomy in the job [27]. Autonomy in a job in terms of the Self-Determination Theory (SDT) is explained as independence, because individuals can undertake acts that affect themselves [27]. Individuals do not derive satisfaction or happiness purely from the outcomes, but by what the outcomes achieve as autonomy is considered as a buffer against the strain of work [4,11,12,28]. According to Atienza et al. [16], job control or autonomy indicates a stronger association with intrinsic job satisfaction. Employee-owned companies report higher levels of job satisfaction, which may be derived from a greater sense of autonomy among the employees [29,30]. Bianchi [13] compares the utilities of employees and entrepreneurs and finds that the non-pecuniary dimensions of job satisfaction, particularly autonomy, have an effect on the difference in utility between the two groups. Entrepreneurs are assumed to be happier because they have a higher level of autonomy than employees [12,13,14]. 

Since psychological needs or intrinsic motivation are considered to be closely related to happiness, the hierarchy of need theory suggests that self-actualization represents the highest level of intrinsic motivation for work and is derived from the desire for self-fulfillment [31]. According to Maslow, self-actualization is recognized as the goal of life, or final happiness, in Aristotle’s value system of happiness [31]. Happiness can be achieved through personal expressiveness when individuals fully involve themselves in activities and fulfill their potentials, especially the involvement in the occupation [32]. A sense of consciousness toward their work and an attitude that gives meaning to life and activities as a worker is also called occupational calling [33,34,35]. Workers higher in occupational calling are typically dedicated to the achievement of their work goals [33,34,35]. The job has become a way to fulfill the potential and achieve self-actualization. The SDT theory also suggests that self-achievement of job is based on basic psychological needs that include competence, relatedness, and autonomy, thus individuals are more likely to realize their abilities, influence, and potential when they have a higher level of autonomy in work [4,16,27]. Entrepreneurs have been found to have a higher level of autonomy than others, and hence are more likely to fulfill their potential and pursue happiness when they are responsible for running businesses [36].

Moreover, Adams [37] identifies the psychological factor of perceived equity as associated with job-related wellbeing and illustrates that this is derived from the comparison between effort and reward in work. When individuals compare effort and reward within or outside an organization, they may feel anger, hatred, or perceive a lack of dignity if they see that their reward is lower than their effort [37]. Conversely, they may feel guilty if they perceive the reward to be far beyond the effort made [37]. Similarly, the Effort-Reward Imbalance model (ERI model) based on a reciprocal relationship between effort and rewards at work [38]. The outcomes suggested that when there is a mismatch between the two, low levels of wellbeing will necessarily result [38]. According to Walster et al. [39], a high level of perceived inequity is accompanied by a high level of depression among workers. Individuals may either distort the input-output ratio or leave an organization if there is a mismatch between effort and reward [40]. Efforts represent job demands or obligations that are imposed on the workforce; while rewards which are distributed both by the employer and society at large consist of money, esteem, job security, and career opportunities [41]. Both employees and entrepreneurs may experience a mismatch between effort and reward, but entrepreneurs have more control over a match within an organization than employees.

Thus, the following hypothesis is proposed:

**Hypothesis** **H1.** *Entrepreneurs are more likely to have high job satisfaction than employees.*

Subjective wellbeing represents an ongoing state of psychological wellness [42], which has been a significant index in research on working conditions [4,13,16,27]. It is considered that subjective wellbeing encompasses both short-term affect and the broader cognitive assessments of an individual’s life [43]. With concern of broaden life boundary, subjective wellbeing can be divided into overall happiness and component happiness [44]. Work, as a significant component in life, has importance in judgments of happiness and wellbeing. Individuals’ dispositions can influence how they gather and recall information about their jobs, while job satisfaction can affect subjective wellbeing because work is often central to the lives of individuals [45]. In their meta-analytic review, Raza et al. [46] found that the average correlation between job and life satisfaction to be high. Thus, a similar hypothesis is proposed as follows:

**Hypothesis** **H2.** *Entrepreneurs are more likely to have a high level of subjective wellbeing than employees.*

According to Leung et al. [6], wellbeing of labor force should be separated into physical and psychological wellbeing. As subjective wellbeing and job satisfaction are measuring psychological wellbeing of labor force, physical wellbeing of labor force is measured by physical health. Physical wellbeing is highly correlated with subjective wellbeing and job satisfaction because numerous studies report a significant reciprocal relationship between physical wellbeing and psychological wellbeing [47]. Oates et al. [48] show that psychological wellbeing may be a protective factor in health, reducing the risk of chronic disease and promoting longevity. According to Judge and Locke [45], unhealthiness often result from negative views that are influenced by psychological states. For instance, rates of depression and heart disease are commonly linked [49,50]. Concerning importance of the physical wellbeing, it is framed as an important indicator of wellbeing or happiness for labor force. The factors which are expected to increase psychological wellbeing of labor force possibly increase physical wellbeing as well. Thus, the following hypothesis about physical wellbeing is proposed:

**Hypothesis** **H3.** *Entrepreneurs are more likely to have a high level of physical wellbeing than employees.*

### 1.2. The Motives of Entrepreneurs Affect Their Happiness

Although entrepreneurs are often treated as a homogeneous group of actors in the literature, they can be very different. Conventional entrepreneurship aims to create wealth [51,52], and achieve competitive advantages [53]. On the contrary, subsistence entrepreneurship is fundamentally about survival [54], and individuals involved in it often lack experience in deploying the cognitive skills needed to discern, evaluate, and exploit growth-oriented opportunities [55]. The conventional entrepreneurial process, which includes entrepreneurial alertness, opportunity recognition and exploitation, and growth decisions [54,55], can be regarded as transformational entrepreneurship. Subsistence entrepreneurs run businesses to provide subsistence income, while transformational entrepreneurs aim to create businesses that can grow far beyond the scope of individual needs [18]. The motive for subsistence entrepreneurs is necessity rather than choice [19], while transformational entrepreneurs are motivated by non-pecuniary benefits [14]. Subsistence entrepreneurs may prefer salaried employment to business ownership but do not have access to salaried work [56]. Subsistence entrepreneurs have lower comparative advantages and abilities than transformational entrepreneurs, who manage their businesses using the skills or resources they possess [53,56].

The differences in economic objectives and skills lead to different levels of satisfaction for entrepreneurs. Donovan [56] concludes that subsistence entrepreneurs often get stuck in unproductive or small firms and achieve lower profits than transformational entrepreneurs. Carree and Verheul [14] state that entrepreneurs motivated by non-pecuniary benefits would probably be less disappointed by unexpected financial hardship, unforeseen stress, or excessively long working hours. Cooper and Artz [57] find that non-monetary goals relate positively to satisfaction. According to Jamal [20], entrepreneurs may experience discomfort when they have no better choice than to become self-employed, and subsistence entrepreneurs who give up the free will associated with jobs may experience a loss of subjective wellbeing [58]. According to Schoar [18], substantial entrepreneurs in China are pushed into self-employment rather than choosing it themselves. Chinese subsistence entrepreneurs generally have lower levels of abilities and gain little non-pecuniary benefits. Chinese subsistence entrepreneurs get stuck in this particular career path, do not fully appreciate it, and thus have lower job-related wellbeing than other entrepreneurs. Thus, the following hypotheses are proposed:

**Hypothesis** **H4.** *Transformational entrepreneurs are more likely to have a high level of job satisfaction than subsistence entrepreneurs.*

**Hypothesis** **H5.** *Transformational entrepreneurs are more likely to have a high level of subjective wellbeing than subsistence entrepreneurs.*

**Hypothesis** **H6.** *Transformational entrepreneurs are more likely to have a higher level of physical wellbeing than subsistence entrepreneurs.*

## 2. Materials and Methods

### 2.1. Data Sources and Sample Composition

The data were obtained from the China Labor-Force Dynamic Survey (CLDS) in 2016, which was funded by “985 Program” of Sun Yat-Sen University and conducted by the Center for Social Survey. CLDS is a multi-purpose survey designed to provide a regular assessment of the living conditions and the quality of life of the Chinese labor force population. About one-third of the survey sample is rotated in the survey in the next round of surveying, which is every two years CLDS were obtained through a stratified three-stage (districts/counties-villages/communities-households) probability random sampling procedure. During all stages of data collection, the research team adopted a face interview, field check, audio record check, interview reviews, and statistical analyses to ensure data quality. The data were released to the researchers without access to any personal data. The individual-level data and survey documentation in 2016 which are available for research (on the website http://css.sysu.edu.cn).

As the age of retirement in China was expected to be 65, individuals aged 15–64 were selected for the survey. In 2016, CLDS covered 29 regions and cities, which included 401 communities, 14,226 families, and 21,086 individuals. Information on the respondents’ living conditions, family situations, education, labor market participation, health, and various aspects of subjective wellbeing were collected through questionnaires. Individuals who clearly stated their working status as entrepreneurs, or employed fulltime, were chosen for the sample. Excluding farmers and those with missing information, a final sample of 8183 was obtained, including 6108 employees and 2075 entrepreneurs.

### 2.2. Variables and Definitions

#### 2.2.1. Dependent Variables

The three main dependent variables used to assess the difference between levels of job-related wellbeing were job satisfaction, subjective wellbeing, and physical wellbeing. Job satisfaction is measured by the question “In general, are you satisfied with your current job?” (1 = very unsatisfied to 5 = very satisfied). It was confirmed that the minimum reliability of the single-item measure for job satisfaction was high, and all correlational tests for validity of the measure were significant [58]. Subjective wellbeing was measured by the question “In general, how happy are you with life?” (1 = very unhappy to 5 = very happy), which was the most commonly measurement used by surveys, such as the World Values Survey. The measurement of subjective wellbeing was found to have moderate reliabilities by undertaking test-retest correlation analysis [59]. Physical wellbeing was measured by self-rated health (SRH) with the question “In general, do you think you are healthy?” (1 = very unhealthy to 5 = very healthy), and SRH was proven a valid and reliable indicator of morbidity and mortality [60].

#### 2.2.2. Independent Variables

As job characteristics and choice influence job-related wellbeing, a few significant independent variables were chosen. In the first step of the study, the researcher checked whether entrepreneurs had higher levels of job-related wellbeing with higher level of autonomy, self-actualization, and perceived equity. So the independent variables included job type, degree of autonomy, degree of self-actualization, and degree of perceived equity. The job type variable was classified as 0 = “employed” and 1 = “entrepreneur.” Degree of autonomy was measured by the three aspects of working content, intensity, and schedule, and each was scored using “1 = decided totally by others,” “2 = decided partly by self,” and 3 = “decided totally by self.” The scales was developed from Breaugh’s 3 types of autonomy, and the Cronbach’s Alpha reached 0.932 [61]. The degree of autonomy was achieved by adding the scores for each aspect. The degree of self-actualization was measured by “how much do you agree that the main purpose of the current job is self-actualization?” (1 = totally disagree to 5 = totally agree), it was proven that self-reference scale of self-actualization has high reliability and validity [62]. Perceived equity was measured by the question “In general, is your achievement equal to your effort” (1 = very unequal to 5 = very equal), which was developed from self-reported effort-reward ratio by Siegrist et al. with high reliability [63]. The motives for entrepreneurship replaced job type and variables of job characters in the second step of the study. The reason for entrepreneurship was obtained by asking the following question “Why did you get involved in this start-up of business? Is it because you had no better choices of work (subsistence entrepreneurship), or because you took advantage of a business opportunity (transformational entrepreneurship), or because of both reasons mentioned above (synthesized entrepreneurship)?” The motives for entrepreneurship were measured using dummy variables for each of the three types of entrepreneurship, including transformational entrepreneurship (0 = “not transformational entrepreneur;” 1 = “transformational entrepreneur”), synthesized entrepreneurship (0 = “not synthesized entrepreneur;” 1 = “synthesized entrepreneur”), and subsistence entrepreneurship (both transformational entrepreneurship and synthesized entrepreneurship are valued as “0”).

To reduce potential confounding effects, several variables commonly thought to correlate with job-related wellbeing were controlled for both steps of the study. The personal characteristics were measured by a region variable (0 = rural, 1 = urban) [22], a gender variable (0 = female, 1 = male) [22,25], an age group variable (1 = 20 or below, 2 = 21–30, 3 = 31–40, 4 = above 40) [22], marital status (0 = unmarried, 1 = married) [22], education (1 = college or below, 2 = bachelor, 3 = master, 4 = doctor) [3,23], working hours per week (1 = 20 h or below, 2 = 21–40 h, 3 = 41–60 h, 4 = 61–80 h, 5 = above 80 h) [3,24,29], annual income (1 = RMB 10,000 or below, 2 = RMB 10,001–50,000, 3 = RMB 50,001–100,000, 4 = RMB 100,001–150,000, 5 = RMB 150,000 or above) [22,25,29], and industry variables which are consist of 9 type of industries, including civil service(reference group in the first step), farming/mining(0 = no, 1 = yes), manufacturing(dummy variable 0 = no, 1 = yes in the first step, reference group in the second step), power supply/construction/geological exploration/hydraulic management(0 = no, 1 = yes), transportation and network(0 = no, 1 = yes), whole/retail/restaurant(0 = no, 1 = yes), real state/finance(0 = no, 1 = yes), science/education/arts/health/media/social service(0 = no, 1 = yes), and others(0 = no, 1 = yes) [23,24,29].

### 2.3. Statistical Analysis

All questionnaire data were input into a computer and analyzed with STATA 14 (StataCorp, college station, TX, USA). Descriptive statistics and relative t-test were computed for the sample. Concerning most of the variables are categorical variables, Chi-square tests were performed for univariable analysis to determine whether there is a statistically significant difference between the expected frequencies, and ordinal logistic regression models were used for multivariable analysis (significance level set at *p* < 0.05). The hypotheses were certified by results of odds ratio in different ordinal logistic regression models. In the first step, when the odds ratio of job type is significantly above 1, the hypotheses were confirmed that entrepreneurs were more likely to have high job-related wellbeing. In the second step, the hypotheses about transformational entrepreneurs were more likely to have high job-related wellbeing than subsistence entrepreneurs if the odds ratio of transformational entrepreneurship is significantly greater than 1.

## 3. Results

Table 1 shows the frequency and distribution of the characteristics of the number of participants in the full sample. Among the 8183 individuals including 2075 entrepreneurs, 1325 were subsistence entrepreneurs, 515 were transformational entrepreneurs, and 235 were synthesized entrepreneurs. 54.3% were from urban areas, 56.4% were male, 82.3% were married, 88% were in college or below, 57.8% were working over 40 h per week, and 53% were over 40 years old. Approximately 72.4% of people finished college education or less. The annual income of most residents was “RMB 10,001–50,000”. Most of them are from science/education/arts/health/media/social service industry (20.6%), and manufacturing industry (19.6%). The majority were lowest of autonomy (29.7%), medium of autonomy (24.7%), and highest of autonomy (25.9%). 47.5% of people agree that the main purpose of the current job is self-actualization, and 46.8% of individuals felt that the achievement equal effort. Approximately 56.3% were satisfied and very satisfied with their jobs, 64.7% were healthy and very healthy, and 65.8% of people rated their happiness as happy or very happy.

Table 2 reveals the frequency and distribution of the characteristics of two subsamples. There exists difference in the distribution of characteristics between employee group and entrepreneur group. For instance, among the employees, 54.3% were males, nearly 15% received bachelor education or above, 24% were from manufacturing industry, 37% reported the lowest degree of autonomy, 56.8% were satisfied and very satisfied with their jobs, 66.6% rated happy or very happy, and 68.8% felt healthy and very healthy; while in the subgroup of entrepreneurs, 62.7% were males, only 3% attained bachelor education or above,36.8% were industry whole/retail/restaurant,8.3% voted for lowest degree of autonomy, 54.6% were satisfied and very satisfied with their jobs, 63.8% felt happy and very happy, and 52.9% rated healthy and very healthy. The difference in the distribution of characteristics between employee group and entrepreneur group is confirmed at significance level 0.05.Specifically, the average degree of job satisfaction (M = 3.43, SD = 0.953), subjective wellbeing (M = 3.78, SD = 0.94), and physical wellbeing (M = 3.23, SD = 1.401) in entrepreneurs appeared to be lower than average degrees of job satisfaction (M = 3.55, SD =0.757), subjective wellbeing (M = 3.86, SD = 0.894), and physical wellbeing (M = 3.86, SD = 0.853) inemployees. As the average degrees of autonomy (M = 5.12, SD = 1.997) and perceived equity (M = 3.08, SD = 1.222) in employees were lower than average degrees of autonomy (M = 7.95, SD = 1.89) and perceived equity (M = 3.17, SD = 1.17) in entrepreneurs, there is no significant differences in the average degree of self-actualization between employees and entrepreneurs. Employees (M = 2.7, SD = 0.961) worked fewer hours per week than entrepreneurs (M = 2.94, SD = 1.283), and employees (M = 2.15, SD = 0.797) earned lower average annual incomes than entrepreneurs (M = 2.22, SD = 1.026). The average age of employees (M = 3.21, SD = 0.906) is younger than the one of entrepreneurs (M = 3.45, SD = 0.755). There was significant difference in education between groups as well. The average education received by employees (M = 1.16, SD = 0.403) was higher than that for entrepreneurs (M = 1.06, SD = 0.212).

In the first step of the research, the aim was to confirm which job type had a higher level of job-related wellbeing. Table 3 displays univariable analysis of job-related wellbeing among people with different characteristics. Job type, autonomy, self-actualization, and perceived equality, all had impacts on participants’ job satisfaction, subjective wellbeing, and physical wellbeing (*p* < 0.05). To further confirm relationship between job type and job-related wellbeing, multivariate analysis is needed.

Since there may exist mediation effects, the multivariate analysis is undertaken in three steps: first of all, all the variables are included except the job type; secondly, the job type is added into analysis; lastly, three interaction terms are added in the analysis, including job type multiplied by autonomy, job type multiplied by self-actualization, and job type multiplied by perceived equality. Table 4, Table 5, Table 6 show the odds ratios of job-related wellbeing by different resident characteristics in the full sample by steps. In the first step in Table 4, autonomy (OR = 1.041; 95% CI = 1.021–1.061), self-actualization (OR = 1.978; 95% CI = 1.88–2.078), and perceived equity (OR = 1.484; 95% CI = 1.43–1.54) were significantly related to increased odds of high job satisfaction with an approximately positive correlation. In the second step of Table 4, the job satisfaction of entrepreneurs was lower than that of employees (OR = 0.629; 95% CI = 0.554–0.714), although autonomy (OR = 1.082; 95% CI = 1.059–1.107), self-actualization (OR = 1.977; 95% CI = 1.882–2.077), and perceived equity (OR = 1.488; 95% CI = 1.434–1.543) were still significantly related to increased odds of high job satisfaction. Holding other variables constant, the odds of high job satisfaction for entrepreneurs (job type = 1) over the odds of high job satisfaction for employees (job type = 0) is 0.629. That is to say, the probability of high job satisfaction for entrepreneurs is 37.1% lower than the one for employees. When three interaction terms were added into model, entrepreneurs still showed a lower probability of high job satisfaction, and only the interaction term job type with self-actualization was significantly related to increase odds of high job satisfaction. Holding other variable constant, the odds of high job satisfaction for entrepreneurs with high degree of self-actualization were 17% higher than the one for employees with high degree of self-actualization. The result shows that entrepreneurs (job type = 1) constantly had lower probability than employees to have high job satisfaction. Thus, H1 was rejected.

Table 5 shows the result when the dependent variable was replaced by subjective wellbeing. The effect of autonomy, self-actualization, and perceived equity on subjective wellbeing was significant in all the models. The higher the resident’s autonomy (OR = 1.087; 95% CI = 1.06–1.115), self-actualization (OR = 1.897; 95% CI = 1.793–2.007), and perceived equity (OR = 1.498; 95% CI = 1.437–1.561) were, the more likely they were to have high subjective wellbeing in the complete model. However, individuals who own businesses were more likely than the employed to have low levels of subjective wellbeing. In step 2, holding other variables constant, the odds of high subjective wellbeing for entrepreneurs (job type = 1) over the odds of high job satisfaction for employees (job type = 0) is 0.798. In step 3, only term job type multiplied with perceived equality was significantly related with the odds of high subjective wellbeing. The odds of high subjective wellbeing for entrepreneurs with high degree of perceived equality were 12% higher than the one for employees with high degree of perceived equality. While entrepreneurs (job type = 1) constantly had lower probability than employees to have high subjective wellbeing (OR = 0.455, 95%CI = 0.265–0.780), and the odds of high subjective wellbeing for entrepreneurs were 54.5% lower than the one for employees. Thus, H2 was rejected.

In Table 6, the dependent variable was replaced with physical wellbeing. In the first step in Table 6, self-actualization (OR = 1.227; 95% CI = 1.174–1.282), and perceived equity (OR = 1.145; 95% CI = 1.107–1.184) were significantly related to increased odds of high physical wellbeing with an approximately positive correlation. On contrary, autonomy (OR = 0.954; 95% CI = 0.936–0.971) was negatively correlated to increased odds of high physical wellbeing. In the second step of Table 6, result shows that entrepreneurs were more likely to have low levels of physical wellbeing (OR = 0.464; 95% CI = 0.410–0.524). Autonomy had no significant influence on physical wellbeing. Self-actualization (OR = 1.219; 95% CI = 1.167–1.274) and perceived equity (OR = 1.145; 95% CI = 1.107–1. 1.185) were positively associated with odds of high physical wellbeing. In the complete model of Table 6, the autonomy (OR = 1.029; 95% CI = 1.005–1.053), self-actualization (OR = 1.16; 95% CI = 1.103–1.220), and perceived equality (OR = 1.181; 95% CI = 1.136–1.226) had significant positive influence on increasing odds of high physical wellbeing. Three interaction terms were also significantly related to increased odds of high physical wellbeing, but only term job type multiplied by self-actualization (OR = 1.219; 95% CI = 1.102–1.348) was positively correlated with the odds of high physical wellbeing. The odds of high physical wellbeing for entrepreneurs with high degree of self-actualization were 21.9% higher than the one for employees with high degree of self-actualization. The other two interaction terms were negatively correlated with the odds of high physical wellbeing. The probability of high physical wellbeing for entrepreneurs with high degree of autonomy was 6.7% lower than the one for employees with high degree of autonomy, and the probability of high physical wellbeing for entrepreneurs with high degree of perceived equality was 13.5% lower than the one for employees with high degree of perceived equality. The job type had no significant influence on physical wellbeing. Thus, H3 was not supported. Entrepreneurs were therefore not found to be more likely to have high job-related wellbeing than employees.

In the second stage of the study, job type and related variables were replaced with two dummy variables for three categories of the motive for entrepreneurship, including transformational entrepreneurship (0 = “not transformational entrepreneur;” 1 = “transformational entrepreneur”), synthesized entrepreneurship (0 = “not synthesized entrepreneur;” 1 = “synthesized entrepreneur”), and subsistence entrepreneurship (both transformational entrepreneurship and synthesized entrepreneurship are valued as “0”). Subsistence entrepreneurship was the reference group among three categories of the motive for entrepreneurship. Since none of entrepreneurs were from civil service sector, the manufacturing industry became the reference category among the different categories of industry. Table 7 presents univariable analysis of job-related wellbeing among entrepreneurs with different characteristics. Only transformational entrepreneurship and annual income had impacts on all participants’ job-related wellbeing (*p* < 0.05). Region and marital status only had influence on subjective wellbeing (*p* < 0.05). Synthesized entrepreneurship and age only had influence physical wellbeing, while industry had impact on both job satisfaction and physical wellbeing (*p* < 0.05).

Table 8 shows the odds ratios of job-related wellbeing by different resident characteristics in the subsample entrepreneur. The result shows that transformational entrepreneurs were more likely than subsistence entrepreneurs to have high levels of job satisfaction (OR = 1.643; 95% CI = 1.335−2.023). In other words, the odds of high job satisfaction for transformational entrepreneurs were 1.643 times that for subsistence entrepreneurs. In contrast, there was no significant difference in the probability of high job satisfaction between synthesized entrepreneurs and subsistence entrepreneurs (*p* = 0.168). Annual income was significantly related to increased odds of high job satisfaction (OR = 1.456, 95% CI = 1.243–1.479). Entrepreneurs with low annual income were less likely to have high job satisfaction than those with high annual income. In that way, transformational entrepreneurs are more likely to have high level of job satisfaction than subsistence entrepreneurs. H4 was therefore confirmed.

The similar result is shown on the odds ratio of subjective wellbeing in Table 8. The chance of high subjective wellbeing for synthesized entrepreneurs was not different significantly from that for subsistence entrepreneurs (*p* = 0.163). Holding other variables constant, the odds of high subjective wellbeing for transformational entrepreneurs over the odds of high subjective wellbeing for subsistence entrepreneurs is 1.662. That was to say, the probability of high subjective wellbeing for transformational entrepreneurs was 66.2% higher than the one for subsistence entrepreneurs. Annual income was significantly related to increased odds of high subjective wellbeing (OR = 1.115, 95% CI = 1.026–1.212) with an approximately positive correlation. Thus H5 was supported by transformational entrepreneurs were more likely to have high level of subjective wellbeing compared to subsistence entrepreneurs.

The odds ratios of physical wellbeing with various factors for entrepreneur group were indicated in the Table 8 as well, in contrast to results of Table 7, transformational and synthesized entrepreneurship did not have significant influence on odds of high physical wellbeing. In addition, age (OR = 0.803; 95% CI = 0.717–0.899) was negatively associated with odds of high physical wellbeing, while annual income (OR = 1.226; 95% CI = 1.13–1.329) were positively associated with odds of high physical wellbeing. Thus H6 was rejected.

To explore the possible reasons why subsistence entrepreneurship decrease the odds of high job-related wellbeing, one-way ANOVA was undertaken to compare the difference of some important characters between the employees and three categories of entrepreneurs in Table 9. Despite a moderate degree of autonomy, the degree of self-actualization and perceived equity were lowest in subsistence entrepreneurs. Compared to employees and other entrepreneurs, subsistence entrepreneurs earned the lowest average level annual income but worked for longer hours per week than employees. Therefore, subsistence entrepreneurs may underestimate the meaning and reward of their jobs to rate the lowest average degree of self-actualization and perceived equality. In addition, the average age of subsistence entrepreneurs was higher than other entrepreneurs and employees, but the average level of education received by subsistence entrepreneurs was lower than that of employees and other entrepreneurs. In that way, subsistence entrepreneurs may have little employment choice due to little comparative advantages. To sum up, subsistence entrepreneurship was negatively associated with odds of high job satisfaction and subjective wellbeing for entrepreneurs [18,20,49,51], which made employees happier in Chinese labor force.

## 4. Discussion

In China, employees are expected to face harsh working conditions and low job-related wellbeing. Individuals therefore substantially rethink their career paths and consider being their own bosses. Entrepreneurs who make business decisions on their own have higher levels of autonomy, self-actualization, and perceived equity than employees who don’t make business decisions on their own. Most research implies that with higher autonomy, self-actualization, and perceived equity, the job-related wellbeing of entrepreneurs will be higher than that of employees [4,14,27,35]. The hypotheses developed and tested study draw on wellbeing related theory and suggest that the impact of entrepreneurship on job-related prosperity varies based on the types of motives.

Using an individual survey across 29 provinces and cities in China, the outcome demonstrated in this study is contrary to previous research. First, despite a moderate degree difference in autonomy and perceived equity, there was no significant difference in self-actualization between employees and entrepreneurs. Although the average degree of perceived equity among entrepreneurs was greater than that of employees, the average differences between both scales of employees and entrepreneurs are nuanced. Second, entrepreneurs were found to be less satisfied, unhappier, and unhealthier than employees, although entrepreneurs had higher levels of autonomy and perceived equality. The results show that autonomy, self-actualization, and perceived equity were positively correlated to increased odds of high job-related wellbeing, while entrepreneurs were less likely to have high level of job-related wellbeing than employees. Third, the interaction terms were added into analysis as well, including job type multiplied with autonomy, job type multiplied with self-actualization, and job type multiplied with perceived equality. The odds of high job satisfaction for entrepreneurs with high degree of self-actualization were 17% higher than the one for employees with high degree of self-actualization; the odds of high subjective wellbeing for entrepreneurs with high degree of perceived equality were 12% higher than the one for employees with high degree of perceived equality; the odds of high physical wellbeing for entrepreneurs with high degree of self-actualization were 21.9% higher than the one for employees with high degree of self-actualization; the probability of high physical wellbeing for entrepreneurs with high degree of autonomy was 6.7% lower than the one for employees with high degree of autonomy; and the probability of high physical wellbeing for entrepreneurs with high degree of perceived equality was 13.5% lower than the one for employees with high degree of perceived equality. In general, the results show no evidence for that entrepreneurs were more likely than employees to have high job-related wellbeing despite the positive influence of autonomy, self-actualization, and perceived equality.

To explore the reason why entrepreneurs were less likely to have high job-related wellbeing in China, the type of entrepreneurship was taken into account. Different types of entrepreneurs have different motives for creating ventures. For instance, transformational entrepreneurs are often individuals who have abilities and ambitious goals to seize innovation opportunities, subsistence entrepreneurs run businesses to provide subsistence income due to lack of skills and work opportunities. In developing countries like China, substantial entrepreneurs create new ventures because they have no choice and no better option. Subsistence entrepreneurs have been found to constitute up to 64% of all entrepreneurs in the survey. When the type of entrepreneurship was added into regression for subsample entrepreneur, it turns out that transformational entrepreneurs were more likely than subsistence entrepreneurs to have high levels of job satisfaction (OR =1.643; 95% CI = 1.335–2.023) and subjective wellbeing (OR = 1.662; 95% CI = 1.36–2.032). It is confirmed that subsistence entrepreneurship decreases the odds of high job-related wellbeing in the labor force. Moreover, the degree of self-actualization and perceived equity were lowest in subsistence entrepreneurs. Compared to employees and other entrepreneurs, subsistence entrepreneurs earned the lowest average level annual income but worked for longer hours per week than employees. In addition, the average age of subsistence entrepreneurs was higher than other entrepreneurs and employees, but the average level of education received by subsistence entrepreneurs was lower than that of employees and other entrepreneurs. In that way, subsistence entrepreneurs may have little employment choice due to little comparative advantages. The career choice of the entrepreneur is not always as individuals imagine it to be in China. Individuals should consider twice about their advantages before they chose to be a “boss”. The government and society should provide more help or service for promoting comparative advantage and wellbeing of entrepreneurs.

## 5. Limitations and Contribution

### 5.1. Limitations

The weaknesses of the study were due to disadvantages in the data and the sample. First, the study was a cross-section study of job-related wellbeing based on a self-reported questionnaire, and the data was relatively limited and based on 29 provinces and cities. Concerning on constraints on generalizability, longitudinal data provided more insights into changes in job-related wellbeing with extended entrepreneurship. Second, the regional data and time in the study were limited, so the influence of entrepreneurship within mature markets was not examined in different provinces and cities, such as financial market flexibilities and the role of government. Third, this study adopted to measure transformational entrepreneurship as “taking advantage of a business opportunity”. The full concept should be explored further in the future, for instance, a more complete scale of transformational entrepreneurship should be developed. Ultimately, individual heterogeneity and selection were not fully controlled in detecting the difference between different career statuses. The contrast of individual characteristics among groups of jobs may affect the distribution of psychological states, such as neuroticism or extraversion. Future research should control the personal aspects within and outside a group to rule out the impact of individual characteristics.

### 5.2. Contributions

The study contributes to the research into job-related wellbeing by developing a new framework that draws attention to the motive for entrepreneurship. Based on the job demand control support model, self-determination theory, and effort-reward imbalance model, this study organizes the research framework and provides comparable evidence of job-related wellbeing between entrepreneurs and employees in China. Entrepreneurs are generally considered to be more satisfied or happier than employees in the previous study. Entrepreneurship is valued by both the government and individuals as a great job choice. However, this study notes that the main effect does not turn out as expected. The entrepreneurs were less likely than employees to have high job-related wellbeing despite the positive influence of autonomy, self-actualization, and perceived equality.

To further explore the reason why the entrepreneurs were less likely than employees to have high job-related wellbeing, the study suggests to introduce incentives of entrepreneurship into research framework for entrepreneurs ’wellbeing study. Except physical wellbeing, the study shows significant evidence that transformational entrepreneurs were more likely to have high job satisfaction and subjective wellbeing than subsistence entrepreneurs. The study also reveals that subsistence entrepreneurs who have less comparative advantages may face unbalanced effort and reward in China. The study discusses the career choice of the entrepreneur in China from the perspective of wellbeing, which is a novel research perspective at our best knowledge. Thus the result provides some suggestions for individuals who imagine to be entrepreneurs in China with consideration of incentive.

## 6. Conclusions

In summary, we report a two-step study examining job-related wellbeing for both employees and entrepreneurs based on multiple theoretical background. In the first step, the result shows the entrepreneurs were less likely than employees to have high job-related wellbeing, which are opposite evidence for previous studies. In the second step, incentives of entrepreneurship was introduced into research framework for entrepreneurs ’wellbeing study to explore the possible reasons why entrepreneurs were less likely than employees to have high job-related wellbeing. The results indicated that transformational entrepreneur were more likely to have high job satisfaction and subjective wellbeing than subsistence entrepreneurs who consist of majority of entrepreneurs in the survey. Compared to employees and other entrepreneurs, subsistence entrepreneurs who have less comparative advantages may face unbalanced effort and reward in China. This study organizes the framework of wellbeing study, provides comparable evidence, and find out a novel view of the impact of career choices in China. On one hand, the findings can prompt researchers to extend the scope of their studies to the incentives of entrepreneurship to increase the happiness of the workforce, on the other hand, the study reveals the temporal situation of entrepreneurs, and inspires individuals how to make career choice for being entrepreneurs in China.

## Figures and Tables

**Table 1:** Characteristics of the full sample.

Characteristics		N	Ratio (%)	M(S.D.)
Job type	employee	6108	74.6	N/A
	entrepreneur	2075	25.4	
MFE	employee	6108	74.6	N/A
	subsistence	1325	16.2	
	transformational	515	6.3	
	synthesized	235	2.9	
Region	rural	3742	45.7	N/A
	urban	4441	54.3	
Gender	female	3567	43.6	N/A
	male	4616	56.4	
Age	≤20 years	214	2.6	3.27 (0.877)
	~30 years	1694	20.7	
	~40 years	1940	23.7	
	>40 years	4335	53	
Marital status	unmarried	1452	17.7	N/A
	married	6731	82.3	
Education	college or below	7204	88	1.13 (0.369)
	bachelor	897	11	
	master	77	0.9	
	doctor	5	0.1	
WHPW	≤20	878	10.7	2.76 (1.058)
	~40	2579	31.5	
	~60	2937	35.9	
	~80	1208	14.8	
	>80	581	7.1	
Annual income	≤10,000 RMB	1227	15	2.17 (0.861)
	~50,000 RMB	5087	62.2	
	~100,000 RMB	1463	17.9	
	~150,000 RMB	175	2.1	
	~200,000 RMB	111	1.4	
	>200,000 RMB	120	1.5	
Industry	civil service	379	4.6	N/A
	farming/mining	273	3.3	
	manufacturing	1607	19.6	
	PS/C/GE/HM	1012	12.4	
	transportation/network	540	6.6	
	whole/retail/restaurant	1372	16.8	
	real state/finance	278	3.4	
	S/E/H/M/SE	1686	20.6	
	others	1036	12.7	
Autonomy	3(lowest)	2432	29.7	5.84 (2.325)
	4	381	4.7	
	5	554	6.8	
	6	2024	24.7	
	7	430	5.3	
	8	243	3	
	9(highest)	2119	25.9	
Self-actualization	strongly disagree	326	4	3.58 (0.954)
	disagree	656	8	
	neutral	2214	27.1	
	agree	3888	47.5	
	strongly agree	1099	13.4	
Perceived equality	very unequal	1743	21.3	3.11 (1.209)
	unequal	74	0.9	
	neutral	2241	27.4	
	equal	3830	46.8	
	very equal	295	3.6	
JS	very unsatisfied	219	2.7	3.52 (0.813)
	unsatisfied	476	5.8	
	neutral	2883	35.2	
	satisfied	4055	49.6	
	very satisfied	550	6.7	
SWB	very unhappy	124	1.5	3.84 (0.906)
	unhappy	355	4.3	
	neutral	2317	28.3	
	happy	3309	40.4	
	very happy	2078	25.4	
PWB	very unhealthy	490	6	3.70 (1.056)
	unhealthy	421	5.1	
	neutral	1970	24.1	
	healthy	3490	42.6	
	very healthy	1812	22.1	
	total	8183	100	

Notes: MFE, motives for entrepreneurship; WHPW, working hours per week; JS, job satisfaction; SWB, subjective wellbeing; PWB physical wellbeing; PS/C/GE/HM, power supply/construction geological exploration/hydraulic management; S/E/H/M/SE, science/education/arts/health/media/social service; OIs, other unnamed industries; N/A, not available.

**Table 2:** Characteristics of the employee and entrepreneur subsamples.

Characteristics		Employee			Entrepreneur		
		N	Ratio (%)	M(S.D.)	N	Ratio (%)	M(S.D.)
Region	rural	2593	42.5	N/A	1149	55.4	N/A
	urban	3515	57.5		926	44.6	
Gender	female	2794	45.7	N/A)	773	37.3	N/A
	male	3314	54.3		1302	62.7	
Age	≤20 years	202	3.3	3.21 (0.906)	12	0.6	3.45 (0.755)
	~30 years	1396	22.9		298	14.4	
	~40 years	1439	23.6		501	24.1	
	>40 years	3071	50.3		1264	60.9	
Marital status	unmarried	1220	20	N/A	232	11.2	N/A
	married	4888	80.0		1843	88.8	
Education	college or below	5199	85.1	1.16 (0.403)	2005	96.6	1.04 (0.212)
	bachelor	834	13.7		63	3.0	
	master	71	1.2		6	0.3	
	doctor	4	0.1		1	0.1	
WHPW	≤20	506	8.3	2.7 (0.961)	372	17.9	2.94 (1.283)
	~40	2209	36.2		370	17.8	
	~60	2312	37.9		625	30.1	
	~80	785	12.9		423	20.4	
	>80	296	4.8		285	13.7	
Annual income	≤10,000 RMB	812	13.3	2.15 (0.797)	415	20.0	2.22 (1.026)
	~50,000 RMB	3999	65.5		1088	52.4	
	~100,000 RMB	1038	17.0		425	20.5	
	~150,000 RMB	123	2.0		52	2.5	
	~200,000 RMB	70	1.1		41	2.0	
	>200,000 RMB	66	1.1		54	2.6	
Industry	civil service	379	6.2	N/A	0	0	N/A
	farming/mining	200	3.2		73	3.5	
	manufacturing	1468	24		139	6.7	
	PS/C/GE/HM	693	11.4		319	15.4	
	transportation/network	419	6.9		121	5.8	
	whole/retail/restaurant	608	10		764	36.8	
	real state/finance	256	4.2		22	1.1	
	S/E/H/M/SE	1462	23.9		224	10.8	
	others	623	10.2		413	19.9	
Autonomy	3(lowest)	2258	37.0	5.12 (1.997)	174	8.4	7.95 (1.892)
	4	363	5.9		18	0.9	
	5	521	8.5		33	1.6	
	6	1791	29.3		233	11.2	
	7	353	5.8		77	3.7	
	8	187	3.1		56	2.7	
	9(highest)	635	10.4		1484	71.5	
Self-actualization	strongly disagree	213	3.5	3.58 (0.936)	113	5.4	3.59 (1.008)
	disagree	512	8.4		144	6.9	
	neutral	1672	27.4		542	26.1	
	agree	2931	48.0		957	46.1	
	strongly agree	780	12.8		319	15.4	
Perceived equality	very unequal	1378	22.6	3.08 (1.222)	365	17.6	3.17 (1.17)
	unequal	2	0		72	3.5	
	neutral	1662	27.2		579	27.9	
	equal	2874	47.1		956	46.1	
	very equal	192	3.1		103	5.0	
JS	very unsatisfied	93	1.5	3.55 (0.757)	126	6.1	3.43 (0.953)
	unsatisfied	329	5.4		147	7.1	
	neutral	2212	36.2		671	32.3	
	satisfied	3087	50.5		968	46.7	
	very satisfied	387	6.3		163	7.9	
SWB	very unhappy	88	1.4	3.86 (0.894)	36	1.7	3.78 (0.94)
	unhappy	223	3.7		132	6.4	
	neutral	1732	28.4		585	28.2	
	happy	2484	40.7		825	39.8	
	very happy	1581	25.9		497	24.0	
PWB	very unhealthy	42	0.7	3.86 (0.853)	448	21.6	3.23 (1.401)
	unhealthy	299	4.9		122	5.9	
	neutral	1563	25.6		407	19.6	
	healthy	2780	45.5		710	34.2	
	very healthy	1424	23.3		388	18.7	

Notes: WHPW, working hours per week; JS, job satisfaction; SWB, subjective wellbeing; PWB physical wellbeing; PS/C/GE/HM, power supply/construction geological exploration/hydraulic management; S/E/H/M/SE, science/education/arts/health/media/social service; OIs, other unnamed industries; N/A, not available.

**Table 3:** The univariable analysis of job-related wellbeing with various factors in full sample.

	JS		SWB		PWB	
Characteristics	Chi2	*p*	Chi2	*p*	Chi2	*p*
Job type	144.13	0.000	29.722	0.000	1200	0.000
Autonomy	238.673	0.000	102.84	0.000	633.293	0.000
Self-actualization	1900	0.000	482.159	0.000	251.7656	0.000
Perceived equality	1400	0.000	951.747	0.000	1500	0.000
Region	12.391	0.015	5.554	0.235	33.745	0.000
Gender	8.229	0.084	15.217	0.004	8.834	0.065
Age	33.702	0.001	17.017	0.149	331.655	0.000
Marital status	12.033	0.15	82.603	0.000	97.047	0.000
Education	53.08	0.000	80.015	0.000	77.712	0.000
WHPW	161.224	0.000	90.637	0.000	188.927	0.000
Annual income	216.228	0.000	87.973	0.000	137.704	0.000
Industry	181.209	0.000	127.719	0.000	395.595	0.000

Notes: WHPW, working hours per week; JS, job satisfaction; SWB, subjective wellbeing; PWB physical wellbeing.

**Table 4:** Ordinal logistic regression of job satisfaction in full sample by steps.

		Step 1				Step 2				Step 3			
	Characteristics	O.R.	*p*-Value	[95% C.I.]		O.R.	*p*-Value	[95% C.I.]		O.R.	*p*-Value	[95% C.I.]	
Step 1	Autonomy	1.041	0.000	1.021	1.061	1.082	0.000	1.059	1.107	1.087	0.000	1.06	1.115
	Self-actualization	1.978	0.000	1.88	2.078	1.977	0.000	1.882	2.077	1.897	0.000	1.793	2.007
	Perceived equality	1.484	0.000	1.43	1.54	1.488	0.000	1.434	1.543	1.498	0.000	1.437	1.561
	Region	0.871	0.003	0.794	0.955	0.843	0.000	0.807	0.971	0.844	0.000	0.769	0.927
	Gender	0.865	0.002	0.789	0.949	0.886	0.010	0.774	0.924	0.885	0.010	0.807	0.971
	Age	1.094	0.002	1.035	1.157	1.116	0.000	1.055	1.181	1.118	0.000	1.057	1.183
	Marital status	0.993	0.913	0.875	1.127	1.008	0.902	0.888	1.144	1.007	0.914	0.887	1.143
	Education	0.952	0.460	0.836	1.085	0.919	0.208	0.806	1.048	0.921	0.219	0.808	1.05
	WHPW	0.95	0.017	0.91	0.991	0.957	0.044	0.919	0.999	0.957	0.044	0.918	0.999
	Annual income	1.254	0.000	1.187	1.325	1.261	0.000	1.193	1.332	1.26	0.000	1.192	1.331
	farming/mining	0.616	0.002	0.451	0.841	0.657	0.008	0.479	0.894	0.65	0.007	0.476	0.887
	manufacturing	0.673	0.001	0.536	0.844	0.681	0.001	0.542	0.854	0.672	0.001	0.536	0.844
	PS/C/GE/HM	0.545	0.000	0.43	0.691	0.595	0.000	0.469	0.755	0.588	0.000	0.463	0.746
	transportation/network	0.596	0.000	0.46	0.774	0.635	0.001	0.489	0.825	0.631	0.001	0.486	0.819
	whole/retail/restaurant	0.621	0.000	0.491	0.785	0.724	0.008	0.57	0.919	0.725	0.008	0.57	0.92
	real state/finance	0.744	0.058	0.549	1.01	0.762	0.080	0.561	1.034	0.757	0.074	0.558	1.027
	S/E/H/M/SE	0.784	0.031	0.629	0.979	0.82	0.079	0.658	1.023	0.816	0.072	0.654	1.018
	OIs	0.553	0.000	0.436	0.701	0.624	0.000	0.491	0.793	0.62	0.000	0.488	0.789
Step 2	Job type					0.629	0.000	0.554	0.714	0.424	0.003	0.24	0.785
Step 3	Job type * Autonomy									0.989	0.996	0.938	1.041
	Job type * Self-actualization									1.173	0.006	1.054	1.306
	Job type * Perceived equality									0.969	0.538	0.889	1.056

Notes: WHPW, working hours per week; JS, job satisfaction; SWB, subjective wellbeing; PWB physical wellbeing; O.R., odds ratio; C.I., confidence interval; PS/C/GE/HM, power supply/construction/geological exploration/hydraulic management; S/E/H/M/SE, science/education/arts/health/media/social service; OIs, other unnamed industries; *, multiplied by.

**Table 5:** Ordinal logistic regression of subjective wellbeing in full sample by steps.

		Step 1				Step 2				Step 3			
	Characteristics	O.R.	*p*-Value	[95% C.I.]		O.R.	*p*-Value	[95% C.I]		O.R.	*p*-Value	[95% C.I.]	
Step 1	Autonomy	1.026	0.007	1.007	1.045	1.046	0.000	1.024	1.068	1.036	0.003	1.012	1.061
	Self-actualization	1.416	0.000	1.355	1.481	1.414	0.000	1.352	1.479	1.426	0.000	1.353	1.503
	Perceived equality	1.383	0.000	1.335	1.433	1.385	0.000	1.336	1.435	1.348	0.000	1.295	1.404
	Region	0.984	0.711	0.901	1.074	0.966	0.446	0.885	1.055	0.965	0.434	0.884	1.054
	Gender	0.853	0.000	0.782	0.931	0.862	0.001	0.790	0.941	0.862	0.001	0.790	0.941
	Age	0.850	0.000	0.806	0.896	0.858	0.000	0.813	0.905	0.857	0.000	0.813	0.905
	Marital status	1.778	0.000	1.574	2.010	1.794	0.000	1.588	2.028	1.786	0.000	1.580	2.018
	Education	1.164	0.014	1.031	1.314	1.146	0.028	1.015	1.294	1.154	0.021	1.022	1.303
	WHPW	0.940	0.002	0.903	0.978	0.943	0.004	0.906	0.981	0.941	0.003	0.904	0.979
	Annual income	1.037	0.161	0.986	1.091	1.038	0.148	0.987	1.092	1.033	0.213	0.982	1.087
	farming/mining	0.889	0.426	0.664	1.189	0.919	0.570	0.687	1.230	0.916	0.553	0.684	1.225
	manufacturing	0.648	0.000	0.523	0.804	0.654	0.000	0.527	0.811	0.652	0.000	0.526	0.809
	PS/C/GE/HM	0.660	0.000	0.527	0.825	0.691	0.001	0.551	0.866	0.700	0.002	0.558	0.877
	transportation/network	0.790	0.060	0.618	1.010	0.817	0.108	0.639	1.045	0.819	0.113	0.641	1.048
	whole/retail/restaurant	0.600	0.000	0.481	0.750	0.649	0.000	0.518	0.814	0.641	0.000	0.511	0.804
	real state/finance	0.795	0.118	0.596	1.060	0.802	0.134	0.601	1.070	0.808	0.147	0.606	1.078
	S/E/H/M/SE	0.767	0.013	0.623	0.945	0.785	0.023	0.637	0.967	0.784	0.022	0.636	0.966
	OIs	0.581	0.000	0.464	0.727	0.618	0.000	0.492	0.774	0.620	0.000	0.494	0.777
Step 2	Job type					0.798	0.000	0.709	0.899	0.455	0.004	0.265	0.780
Step 3	Job type * Autonomy									1.041	0.110	0.991	1.094
	Job type * Self-actualization									0.973	0.580	0.883	1.072
	Job type * Perceived equality									1.122	0.006	1.033	1.219

Notes: WHPW, working hours per week; JS, job satisfaction; SWB, subjective wellbeing; PWB physical wellbeing; O.R., odds ratio; C.I., confidence interval; PS/C/GE/HM, power supply/construction/geological exploration/hydraulic management; S/E/H/M/SE, science/education/arts/health/media/social service; OIs, other unnamed industries; *, multiplied by.

**Table 6:** Ordinal logistic regression of physical wellbeing in full sample by steps.

		Step 1				Step 2				Step 3			
	Characteristics	O.R.	p-Value	[95% C.I.]		O.R.	p-Value	[95% C.I.]		O.R.	p-Value	[95% C.I.]	
Step 1	Autonomy	0.954	0.000	0.936	0.971	1.013	0.208	0.993	1.035	1.029	0.015	1.005	1.053
	Self-actualization	1.227	0.000	1.174	1.282	1.219	0.000	1.167	1.274	1.160	0.000	1.103	1.220
	Perceived equality	1.145	0.000	1.107	1.184	1.145	0.000	1.107	1.185	1.181	0.000	1.136	1.226
	Region	1.068	0.136	0.979	1.165	1.013	0.775	0.928	1.106	1.014	0.753	0.929	1.107
	Gender	1.036	0.421	0.950	1.129	1.072	0.116	0.983	1.169	1.069	0.134	0.980	1.165
	Age	0.677	0.000	0.641	0.714	0.697	0.000	0.660	0.735	0.698	0.000	0.662	0.736
	Marital status	1.054	0.390	0.935	1.187	1.078	0.219	0.956	1.215	1.082	0.199	0.960	1.219
	Education	0.989	0.856	0.878	1.114	0.940	0.315	0.834	1.060	0.934	0.265	0.828	1.053
	WHPW	0.950	0.013	0.913	0.989	0.962	0.056	0.924	1.001	0.963	0.069	0.925	1.003
	Annual income	1.103	0.000	1.048	1.161	1.111	0.000	1.055	1.169	1.116	0.000	1.060	1.175
	farming/mining	0.881	0.394	0.658	1.179	0.981	0.899	0.732	1.315	0.970	0.840	0.724	1.301
	manufacturing	0.780	0.019	0.633	0.961	0.794	0.031	0.645	0.979	0.783	0.022	0.635	0.965
	PS/C/GE/HM	0.812	0.062	0.653	1.010	0.944	0.607	0.757	1.176	0.915	0.433	0.734	1.142
	transportation/network	0.733	0.012	0.577	0.933	0.805	0.078	0.632	1.024	0.792	0.059	0.622	1.009
	whole/retail/restaurant	0.572	0.000	0.460	0.711	0.733	0.006	0.587	0.914	0.742	0.009	0.594	0.927
	real state/finance	0.800	0.122	0.604	1.061	0.828	0.189	0.625	1.098	0.810	0.142	0.610	1.074
	S/E/H/M/SE	0.835	0.081	0.682	1.023	0.893	0.273	0.729	1.094	0.886	0.245	0.723	1.086
	OIs	0.601	0.000	0.483	0.748	0.727	0.005	0.583	0.908	0.722	0.004	0.579	0.901
Step 2	Job type					0.464	0.000	0.410	0.524	0.588	0.057	0.341	1.015
Step 3	Job type * Autonomy									0.933	0.008	0.887	0.982
	Job type * Self-actualization									1.219	0.000	1.102	1.348
	Job type * Perceived equality									0.865	0.001	0.797	0.940

Notes: WHPW, working hours per week; JS, job satisfaction; SWB, subjective wellbeing; PWB physical wellbeing; O.R., odds ratio; C.I., confidence interval; PS/C/GE/HM, power supply/construction/geological exploration/hydraulic management; S/E/H/M/SE, science/education/arts/health/media/social service; OIs, other unnamed industries; *, multiplied by.

**Table 7:** The univariable analysis of job-related wellbeing with various factors in subsample entrepreneur.

	JS		SWB		PWB	
Characteristics	Chi2	*p*	Chi2	*p*	Chi2	*p*
TE	56.125	0.000	37.67	0.000	44.93	0.000
SyE	6.9486	0.139	0.755	0.944	20.778	0.000
Region	3.12	0.538	20.419	0.000	4.624	0.328
Gender	11.393	0.022	6.833	0.145	0.618	0.961
Age	10.543	0.568	9.739	0.639	78.478	0.000
Marital status	10.808	0.213	45.107	0.000	8.404	0.395
Education	11.766	0.465	15.615	0.21	14.485	0.271
WHPW	31.233	0.013	22.527	0.127	30.551	0.015
Annual income	97.921	0.000	43.502	0.002	85.323	0.000
Industry	84.4547	0.000	39.9064	0.474	65.1635	0.007

Notes: TE, transformational entrepreneurship; SyE, synthesized entrepreneurship; WHPW, working hours per week; JS, job satisfaction; SWB, subjective wellbeing; PWB physical wellbeing.

**Table 8:** Ordinal logistic regression of job-related wellbeing with different factors in subsample entrepreneur.

	JS				SWB				PWB			
Characteristics	Odds Ratio	*p*-Value	[95% C.I.]		Odds ratio	*p*-Value	[95% C.I.]		Odds Ratio	*p*-Value	[95% C.I.]	
TE	1.643	0.000	1.335	2.023	1.662	0.000	1.36	2.032	0.992	0.938	0.813	1.211
SyE	1.21	0.168	0.923	1.586	1.201	0.163	0.928	1.553	0.806	0.102	0.622	1.043
Region	0.745	0.001	0.627	0.885	0.792	0.007	0.669	0.937	0.968	0.698	0.821	1.141
Gender	1.034	0.717	0.864	1.236	0.843	0.056	0.708	1.004	0.942	0.491	0.794	1.117
Age	1.134	0.033	1.01	1.274	0.908	0.097	0.811	1.018	0.803	0.000	0.717	0.899
Marital status	0.967	0.810	0.733	1.275	1.901	0.000	1.442	2.507	1.063	0.651	0.816	1.383
Education	1.337	0.158	0.894	2	1.645	0.014	1.107	2.443	0.907	0.603	0.627	1.311
WHPW	1.046	0.178	0.98	1.116	0.975	0.432	0.915	1.039	0.96	0.191	0.902	1.207
Annual income	1.456	0.000	1.243	1.479	1.115	0.010	1.026	1.212	1.226	0.000	1.13	1.329
farming/mining	0.839	0.526	0.488	1.443	1.16	0.576	0.691	1.947	0.964	0.888	0.579	1.604
PS/C/GE/HM	0.671	0.041	0.458	0.984	0.892	0.543	0.617	1.289	1.175	0.380	0.819	1.686
transportation/network	0.561	0.015	0.352	0.893	0.958	0.855	0.609	1.508	0.768	0.249	0.491	1.203
whole/retail/restaurant	0.773	0.149	0.545	1.097	0.839	0.308	0.599	1.176	0.74	0.075	0.532	1.03
real state/finance	0.749	0.497	0.318	1.744	1.774	0.209	0.725	4.338	0.593	0.182	0.275	1.278
S/E/H/M/S	1.291	0.219	0.859	1.941	0.944	0.774	0.636	1.4	0.968	0.87	0.657	1.427
OIs	0.648	0.022	0.447	0.94	0.741	0.101	0.517	1.06	0.889	0.513	0.624	1.265

Notes: TE, transformational entrepreneurship; SyE, synthesized entrepreneurship; WHPW, working hours per week; JS, job satisfaction; SWB, subjective wellbeing; PWB, physical wellbeing; C.I., confidence interval; PS/C/GE/HM, power supply/construction/geological exploration/hydraulic management; S/E/H/M/S, science/education/arts/health/media/social service; OIs, other industries.

**Table 9:** Comparison between the employees and 3 categories of entrepreneurs.

	Employee	SE	TE	SyE	F	Sig.
	M(S.D.)	M(S.D.)	M(S.D.)	M(S.D.)		
Age	3.21(0.906)	3.54(0.702)	3.28(0.818)	3.34(0.819)	54.034	0.000
Education	1.16(0.403)	1.02(0.144)	1.08(0.322)	1.04(0.213)	63.758	0.000
WHPW	2.7(0.961)	2.92(1.297)	2.98(1.232)	2.96(1.317)	28.04	0.000
Annual income	2.15(0.797)	2.02(0.876)	2.71(1.249)	2.25(0.897)	84.68	0.000
Autonomy	5.12(1.997)	7.75(2.06)	8.3(1.483)	8.36(1.488)	1085.871	0.000
Self-actualization	3.58(0.936)	3.48(1.038)	3.89(0.91)	3.59(0.903)	23.254	0.000
Perceived equality	3.08(1.222)	3.05(1.207)	3.43(1.098)	3.34(0.988)	16.941	0.000
JS	3.55(0.757)	3.35(0.939)	3.66(0.902)	3.42(1.06)	17.736	0.000
SWB	3.86(0.894)	3.69(0.951)	3.99(0.888)	3.8(0.918)	202.01	0.000
PWB	3.86(0.853)	3.24(1.348)	3.27(1.488)	3.05(1.489)	28.873	0.000

Notes: TE, transformational entrepreneurship; SE, subsistence entrepreneurship; SyE, synthesized entrepreneurship; WHPW, working hours per week; JS, job satisfaction; SWB, subjective wellbeing; PWB, physical wellbeing.

## Data Availability

Data available on request due to restrictions of privacy. The data presented in this study are available on request from the Center for Social Survey, Sun Yat-sen University. The data are not publicly available due to policy of Sun Yat-sen University.

## References

[1] Lin Q.Q., Zhong R. (2019). ‘996’ Is China’s Version of Hustle Culture, Tech Workers Are Sick of It. The New York Times.

[2] Crossley G. (2019). China Implementing New Rules to Make Business Easier: State Planner. Reuters.

[3] Sun J., Li X., Lin C. (2016). Development and measurement of subjective wellbeing. Hum. Resour. Dev. China.

[4] Molina-Sánchez H., Ariza-Montes A., Ortiz-Gómez M., Leal-Rodríguez A. (2019). The subjective well-Being challenge in the accounting profession: The role of job resources. Int. J. Environ. Res. Public Health.

[5] Warr P. (1987). Work, Unemployment and Mental Health.

[6] Leung A.S.M., Cheung Y.H., Liu X. (2011). The relations between life domain satisfaction and subjective well-being. J. Manag. Psychol..

[7] Micu A.-E., Necula R.V. (2017). Pursuing happiness in the workplace, purpose and challenge for human resources management. Ovidius Univ. Ann. Ser. Econ. Sci..

[8] Chinese Entrepreneur Survey System (2015). What kind of Entrepreneurs are happier—Review of survey on Chinese entrepreneur happiness for ten years. Econ. Aff..

[9] Wu T.J., Yuan K.S., Yen D.C., Xu T. (2019). Building up resources in the relationship between work–family conflict and burnout among firefighters: Moderators of guanxi and emotion regulation strategies. Eur. J. Work Organ. Psychol..

[10] Wu T.J., Wang L.Y., Gao J.Y., Wei A.P. (2020). Social support and well-being of Chinese special education teachers—An emotional labor perspective. Int. J. Environ. Res. Public Health.

[11] Benz M., Frey B. (2004). Being independent raises happiness at work. Swed. Econ. Policy Rev..

[12] Benz M., Frey B.S. (2008). The value of doing what you like: Evidence from the self-employed in 23 countries. J. Econ. Behav. Organ..

[13] Bianchi M. (2012). Financial development, entrepreneurship and job satisfaction. Rev. Econ. Stat..

[14] Carree M.A., Verheul I. (2012). What makes entrepreneurs happy? Determinants of satisfaction among founders. J. Happiness Stud..

[15] Salinas-Jiménez M., Artés J., Salinas-Jiménez J. (2010). Income, motivation, and satisfaction with life: An empirical analysis. J. Happiness Stud..

[16] Atienza F.L., Castillo I., Appleton P.R., Balaguer I. (2020). Examining the mediating role of motivation in the relationship between multidimensional perfectionism and well- and ill-being in vocational dancers. Int. J. Environ. Res. Public Health.

[17] Feldman D.C., Bolino M.C. (2000). Career patterns of the self-employed: Career motivations and career outcomes. J. Small Bus. Manag..

[18] Schoar A. (2010). The divide between subsistence and transformational entrepreneurship. Innov. Policy Econ..

[19] Amóros J.E., Cristi O., Minniti M. (2011). Poverty, human development and entrepreneurship. The Dynamics of Entrepreneurship: Theory and Evidence.

[20] Jamal M. (1997). Job stress, satisfaction, and mental health: An empirical examination of self-employed and non-self-employed Canadians. J. Small Bus. Manag..

[21] Chi C.G., Cai R., Li Y. (2017). Factors influencing residents’ subjective well-being at World Heritage Sites. Tourism Manag..

[22] Xu W., Sun H., Zhu B., Bai W., Yu X., Duan R., Kou C., Li W. (2019). Analysis of factors affecting the high subjective well-being of Chinese residents based on the 2014 China family panel study. Int. J. Environ. Res. Public Health.

[23] Cannas M., Sergi B.S., Sironi E., Mentel U. (2019). Job satisfaction and subjective well-being in Europe. Econ. Sociol..

[24] Broberg P., Tagesson T., Uman T. (2020). Antecedents of psychological well-being among Swedish audit firm employees. Int. J. Environ. Res. Public Health.

[25] Meisenberg G., Woodley M.A. (2015). Gender differences in subjective well-being and their relationships with gender equality. J. Happiness Stud..

[26] Karasek R.A., Theorell T. (1990). Healthy Work: Stress, Productivity and the Reconstruction of Working Life.

[27] Ryan R.M., Deci E.L. (2000). Self-determination theory and the facilitation of intrinsic motivation, social development, and well-being. Am. Psychol..

[28] Meyer S.C., Hünefeld L. (2018). Challenging cognitive demands at work, related working conditions, and employee well-being. Int. J. Environ. Res. Public Health.

[29] Wu T.J., Gao J.Y., Wang L.Y., Yuan K.S. (2020). Exploring links between polychronicity and job performance from the person–environment fit perspective—The mediating role of well-being. Int. J. Environ. Res. Public Health.

[30] McQuaid R., Canduela J., Egdell V., Dutton M., Raeside R. (2013). The Growth of Employee Owned Businesses in Scotland, Final Report to Cooperative Development Scotland. https://dspace.stir.ac.uk/bitstream/1893/22036/1/The%20growth%20of%20employee%20owned%20businesses%20in%20Scotland%2013.pdf.

[31] Zeigler-Hill V., Vrabel J.K., Sauls D., Lehtman M.J. (2019). Integrating motivation into current conceptualizations of personality. Pers. Indiv. Differ..

[32] Waterman A.S. (1993). Two conceptions of happiness: Contrasts of personal expressiveness (eudaimonia) and hedonic enjoyment. J. Per. Soc. Psychol..

[33] Donati S., Zappalà S., González-Romá V. (2020). The double-edge sword effect of interorganizational trust on involvement in interorganizational networks: The mediator role of affective commitment. Eur. Manag. J..

[34] Wilson C.A., Britt T.W. Living to Work: The Role of Occupational Calling in Response to Challenge and Hindrance Stressors. https://www.tandfonline.com/doi/full/10.1080/02678373.2020.1743791.

[35] Christensen P.H., Foss N.J. (2020). Present-but-online: How mobile devices may harm purposeful co-presence in organizations (and what can be done about it). Eur. Manag. J..

[36] Schneck S. (2014). Why the self-employed happier: Evidence from 25 European countries. J. Bus. Res..

[37] Côté K., Lauzier M., Stinglhamber F. (2020). The relationship between presenteeism and job satisfaction: A mediated moderation model using work engagement and perceived organizational support. Eur. Manag. J..

[38] Siegrist J. (1996). Adverse health effects of high-effort/low reward conditions. J. Occup. Health Psychol..

[39] Walster E., Traupmann J., Walster G.W. (1978). Equity and extramarital sexuality. Arch. Sex. Behav..

[40] Cheng B., Zhou X., Guo G., Yang K. (2020). Perceived overqualification and cyberloafing: A moderated-mediation model based on equity theory. J. Bus. Ethics.

[41] Vegchel N.V., Jonge J.D., Bosma H., Schaufeli W. (2005). Reviewing the effort–reward imbalance model: Drawing up the balance of 45 empirical studies. Soc. Sci. Med..

[42] Jebb A.T., Morrison M., Tay L., Diener E. (2020). Subjective well-being around the world: Trends and predictors across the life span. Psychol. Sci..

[43] Howell R.T., Howell C.J. (2008). The relation of economic status to subjective wellbeing in developing countries: A meta-analysis. Psychol. Bull..

[44] Wu T.J., Xu T., Li L.Q., Yuan K.S. (2020). Touching with heart, reasoning by truth! The impact of Brand cues on mini-film advertising effect. Int. J. Advert..

[45] Judge T.A., Locke E.A. (1993). Effect of dysfunctional thought processes on subjective wellbeing and job satisfaction. J. Appl. Psychol..

[46] Raza M.Y., Rafique T., Hussain M.M., Ali H., Mohsin M., Shah T.S. (2015). The impact of working relationship quality on job Satisfaction and sales person performance: An adaptive selling behavior. Asia Pac. J. Manag. Res. Innov..

[47] Wang X., Jia X., Zhu M., Chen J. (2015). Linking health states to subjective well-being: An empirical study of 5854 rural residents in China. Public Health.

[48] Oates J., Jones J., Drey N. (2017). Subjective well-being of mental health nurses in the United Kingdom: Results of an online survey. Int. J. Ment. Health Nurs..

[49] Joynt K.E., Whellan D.J., O’Connor C.M. (2003). Depression and cardiovascular disease: Mechanisms of interaction. Biol. Psychiat..

[50] Steptoe A., Wardle J. (2005). Positive affect and biological function in everyday life. Neurobiol. Aging.

[51] Hitt M.A., Ireland D.R., Sirmon D.G., Trahms C.A. (2011). Strategic entrepreneurship: Creating value for individuals, organizations and society. Acad. Manag. Perspect..

[52] Zahra S.A., George G., Hitt M.A., Ireland R.D., Camp S.M., Sexton D.L. (2002). International entrepreneurship: The current status of the field and future research agenda. Strategic Entrepreneurship: Creating a New Mindset.

[53] Viswanathan M., Rosa J.A., Ruth J.A. (2010). Exchanges in marketing systems: The case of subsistence consumer merchants in Chennai. India. J. Mark..

[54] Viswanathan M. (2007). Understanding product and market interactions in subsistence marketplaces: A study in South India. Adv. Int. Manag..

[55] Webb J.W., Tihanyi L., Ireland R.D., Sirmon D.G. (2009). You say illegal, I say legitimate: Entrepreneurship in the informal economy. Acad. Manag. Rev..

[56] Donovan K. (2014). Subsistence Entrepreneurs and Misallocation. Meeting Papers 771, Society for Economic Dynamics. https://economicdynamics.org/meetpapers/2014/paper771.pdf.

[57] Cooper A.C., Artz K.W. (1995). Determinants of satisfaction for entrepreneurs. J. Bus. Ventur..

[58] Dolbier C.L., Webster J.A., McCalister K.T., Mallon M.W., Steinhardt M. (2005). Reliability and validity of a single-item measure of job satisfaction. Am. J. Health Promot..

[59] Krueger A.B., Schkade D.A. (2008). The reliability of subjective well-being measures. J. Public Econ..

[60] Gries T., Naudé W.A. (2011). Entrepreneurship and human development: A capability approach. J. Public Econ..

[61] Breaugh J.A. (1985). The measurement of work autonomy. Hum. Relat..

[62] Lefrançois R., Leclerc G., Dubé M., Hébert R., Gaulin P. (1997). The development and validation of a self-report measure of self-actualization. Soc. Behav. Personal..

[63] Siegrist J., Starke D., Chandola T., Godin I., Marmot M., Niedhammer I., Peter R. (2004). The measurement of effort-reward imbalance at work: European comparisons. Soc. Sci. Med..

